# Systematic Review: Study of the Prescribing Pattern of Antibiotics in Outpatients and Emergency Departments in the Gulf Region

**DOI:** 10.3389/fphar.2020.585051

**Published:** 2020-12-15

**Authors:** Rana Kamran Mahmood, Syed Wasif Gillani, Muhammad Waqas Saeed, Muhammad Umar Hafeez, Shabaz Muhammad Gulam

**Affiliations:** ^1^ College of Pharmacy, Gulf Medical University, Ajman, United Arab Emirates; ^2^ Department of Pharmacy, Response Plus Medical, Abu Dhabi, United Arab Emirates; ^3^ Department of Pharmacy, Rashid Hospital, Dubai, United Arab Emirates

**Keywords:** antibiotics, prescribing, gulf countries, outpatient, medication

## Abstract

**Purpose:** To study the prescribing pattern of antibiotics in outpatients and emergency departments in the Gulf region. To compare the appropriateness of prescriptions and antibiotics commonly prescribed for respiratory tract infection.

**Method:** The search was limited to the years 2008–2020, and articles had to be in English. Articles were searched from various resources and evaluated using PRISMA. Forty-one articles were selected and screened, and in the end, 17 articles were included in the study. All articles were selected from the gulf region of six countries: UAE, Saudi Arabia, Qatar, Oman, Yemen, and Bahrain. Only primary literature were included. Inpatient and literature from other countries outside the gulf region were excluded.

**Result:** Penicillins, cephalosporins, and macrolides are highly useful antibiotics for respiratory tract infections. Ceftriaxone IV is recommended in acute respiratory tract infection if therapy with penicillin fails. Most of the antibiotic prescriptions in Gulf countries are inappropriate. Inappropriate antibiotic prescribing in the gulf region varies from place to place and reaches a maximum of 80%. Antibiotics may be prescribed with the wrong dosage or frequency and inappropriate guidelines. Penicillins are prescribed at about 50–60%; the most common penicillins prescribed are amoxicillin and co-amoxiclave. Cephalosporins are prescribed at about 30%, and the most common are third-generation. Macrolides are prescribed at about 17–20%, and the most common macrolides are azithromycin and clarithromycin. Fluoroquinolones are prescribed at about 10–12%, of which levofloxacin and ciprofloxacin are more commonly prescribed with metronidazole at 10%.

**Conclusion:** It is suggested that the antibiotic-prescribing pattern in outpatient and emergency departments in the Gulf region are highly inappropriate and need improvement through education, following guidelines, annual vaccination, and stewardship programs; the most prescribed antibiotic is amoxicillin/co-amoxiclave, and the most often encountered infection in outpatients is acute respiratory tract infection.

## Introduction

Antibiotics are commonly prescribed in outpatient and emergency departments and comprise one a major percentage of outpatient prescriptions. If antimicrobial resistance continues to rise through 2050, it is estimated that 10 million people will die every year, the GDP will be reduced between 2 and 3.5%, and this will cost the world about 100 trillion U.S. dollars (O’neill, 2014). There is a rapid global increase in Watch antibiotic consumption (90.9%) compared to Access antibiotics, especially in lower and middle income countries from year 2000–2015 (Klein et al., 2020). In 2015, prescriptions for antibiotics were 269 million; that is equivalent to 838 antibiotic prescriptions per 1,000 people, and they were dispensed from community pharmacies. This shows that outpatients are an important target for improvement (Centers for Disease Control and Prevention, 2015). Antibiotic use selects for antibiotic-resistant bacteria; antibiotic-resistant organisms infect at least two million people, cause at least 23,000 deaths, and result in $20 billion in excess direct healthcare costs in the United States each year (Public Health England, 2014). It is estimated that there is a substantial increase in burden of infections with antibiotic-resistant bacteria as compared to the other infectious diseases in Europe and the European economic area since 2007 (Cassini et al., 2019). A new report from the OECD predicts that, in just Europe, America, and Australia, 2.4 million people will die in the next 30 years due to resistant microorganisms, and this can cost up to 3.5 billion U.S. dollars (Hofer, 2018). Due to rising levels of resistance, the incidence of death and cost of treatment is increasing day by day, and a major source of antibiotic prescribing is from outpatient and emergency departments. *Clostridium difficile* infection is a life-threatening antibiotic-associated ADE. *C. difficile* caused an estimated 450,000 infections and 15,000 deaths in 2011 in the United States (Lessa et al., 2015). Reductions in specific antibiotics, including fluoroquinolones and cephalosporins, are especially impactful in preventing *C. difficile* infection (Dingle et al., 2017). In a systematic review, it is analyzed that antibiotic-resistant ESKAPE pathogens may cause a high cost burden on economics, and risk of mortality will be very high (Founou et al., 2017).

Antibiotic prescribing with respect to the dosage, frequency, and guidelines for appropriate and inappropriate prescription that may cause an increase in resistance and overuse or underuse of medication may cause an increase in cost or decrease in the outcomes of therapy. Among all outpatient antibiotic prescriptions filled by 19,203,264 privately insured U.S. children and nonelderly adults in 2016, 23.2% were inappropriate, 35.5% were potentially appropriate, and 28.5% were not associated with a recent diagnosis code (Chua et al., 2019). The inappropriate numbers of prescriptions are very high. According to one study, 30% of outpatient prescriptions are inappropriate (Fleming-Dutra et al., 2016). Strategies to reduce inappropriate prescriptions, such as this one study, include displaying poster-sized commitment letters in examination rooms about decreased inappropriate antibiotic prescribing for ARIs. The effect of this simple, low-cost intervention is comparable in magnitude to costlier, more intensive quality-improvement efforts (Meeker et al., 2014).

Respiratory tract infection is the most encountered infection in outpatients over all other infections. A study in the United States reveals that antibiotics prescribed from 2000 to 2010 were 1.4 billion. The rate of broad-spectrum antibiotics prescribed doubled in this time frame, but the rate of prescribing antibiotics was reduced (Lee et al., 2014). In another study, it is revealed that macrolide prescribing for acute respiratory tract infection increased from 2005 to 2012 (Jones et al., 2015). To reduce the inappropriate prescribing of medication in acute respiratory tract infection, the detection of *H. Influenza* can help in reduce the prescribing of antibiotics in a large number of patients (Havers et al., 2018). High antimicrobial resistance in URTIs are due to the high rate of over-the-counter purchasing of antibiotics that range from 40% to 70% in different areas of world (Godman et al., 2020).

Antibiotic prescribing in outpatients as well as inpatients is relatively increased in the last few months due to the COVID-19 pandemic. A study illustrates that the prevalence of bacterial/fungal coinfection with COVID-19 is 8% although the use of broad-spectrum antimicrobial therapy is reported as 72% of COVID-19 patients receiving antimicrobials (Rawson et al., 2020). This increase in consumption of antibiotics may cause a disastrous effect on resistance in the future.

### Purpose

The aim of this systemic review is to uncover the prescribing pattern of antibiotics in the Gulf region, the appropriateness of antibiotics as per guidelines, and the antibiotics prescribed for respiratory tract infection.

## Methods

### Search Subjects

In this study, a required criterion is to search the literature for patients with systemic infections that are prescribed at least one antibiotic and study the antibiotic appropriateness as per guidelines and commonly prescribed antibiotics in respiratory tract infections.

### Primary Search

Different databases and individual journal websites, such as Pubmed, Google Scholar, Microsoft Academic, and EMBASE were searched.

### Search Criteria

Studies included in the systematic review had to fulfill the established criteria: published between 2008 and 2020 and in English. All studies of primary literature had to be in the Gulf region consisting of six countries: Saudi Arabia, UAE, Bahrain, Qatar, Yemen, and Oman. Articles were on the prescribing pattern of antibiotics in outpatient and emergency departments of hospitals or clinics. Excluded were articles on inpatient antibiotic-prescribing patterns, studies elsewhere than the Gulf region, antibiotic-adherence studies, and self-medication from the community pharmacy.

### Search Keywords

Search keywords were antibiotics, prescribing patterns, Gulf countries, outpatient, emergency, and medication.

### Secondary Search

A secondary search was focused mainly on reference articles, titles, and abstracts. Articles that passed through primary screening were critically appraised for inclusion in the study analysis.

## Results and Findings

### PRISMA

**FIGURE 1:**
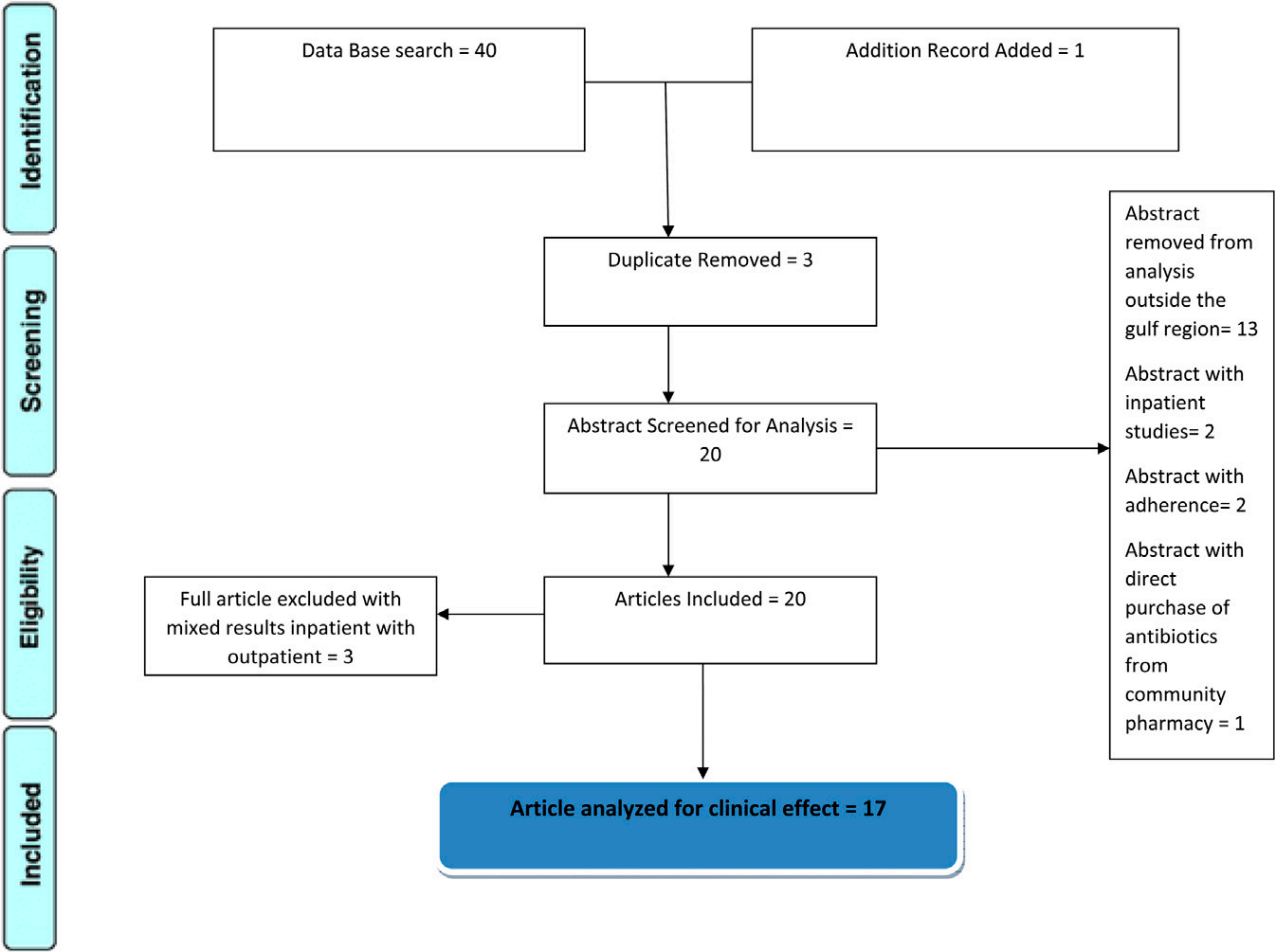
schematic diagram showing the screening and eligibility of articles; 17 are critically analyzed.

As shown in Figure 1 forty articles were found, out of which 3 were duplicates and removed, 13 were excluded for being outside the Gulf region, and two were for inpatients. Three that combined inpatients and outpatients were excluded and a new search added one article; in the end, 17 are included in the study with critical analysis.

#### Treatment Plans for Respiratory Tract Infections in OPAT

John et al. (2014) studied trends of antibiotic prescribing in tonsillitis and found that more than 30.25% of patients were prescribed with co-amoxiclave, 16.8% with ceftriaxone, and 14.7% with metronidazole. As is suggested, cephalosporins alone or in combination with metronidazole when anaerobes are implicated have the highest bacteriological and clinical efficacy (Pichichero, 2006). About 41.2% of antibiotics prescribed are parenteral (John et al., 2014). In another study on outpatient tonsilopharyngitis, Al Alawi et al. (2015) found that ceftriaxone IV gave better results due to the failure of oral antibiotic therapy; 50% of co-amoxiclave failure is due to oral therapy, and patients are shifted to IV ceftriaxone. Senok et al. (2009) studied the antibiotic-prescribing pattern in upper respiratory tract infections in Bahrain and found the order of prescribing as follows: amoxicillin, amoxicillin/clavulanate, cefaclor, erythromycin, cefprozil, and cefuroxime for 5–7 days and azithromycin for 3 days. In another study on prescribing patterns of antibiotics in upper respiratory tract infections, Butt et al. (2017) found that cephalosporins are prescribed about 43%, penicillins and co-amoxiclave 28%, macrolide 19%, flouroquinolones 9%, and 5% of drugs are intravenou. Shaheen et al. (2018) studied the prescribing pattern of antibiotics in OPAT in children for acute respiratory tract infection. The study found that amoxicillin is highly prescribed in 53.3% of cases, the second choice was ceftriaxone in 15.7%, followed by Augmentin in 8.7%, various other antibiotics in 17.5%, and last more than one antibiotic was prescribed in 14/515 cases. El-Gamal et al. (2020) studied management of acute respiratory infection in OPAT. The authors found that the antibiotics most prescribed were penicillin 64.7%, macrolide 23.5%, ceftriaxone 11.7%, and ciprofloxacin 5.9%. Alanazi et al. (2018) studied antibiotic-prescribing patterns in an emergency department in Saudi Arabia and found that the most common antibiotics prescribed in upper respiratory tract infections were penicillins (45%) and then macrolides (35.1%) and cephalosporins 30% (Ahmed, 2020d).

**FIGURE 2:**
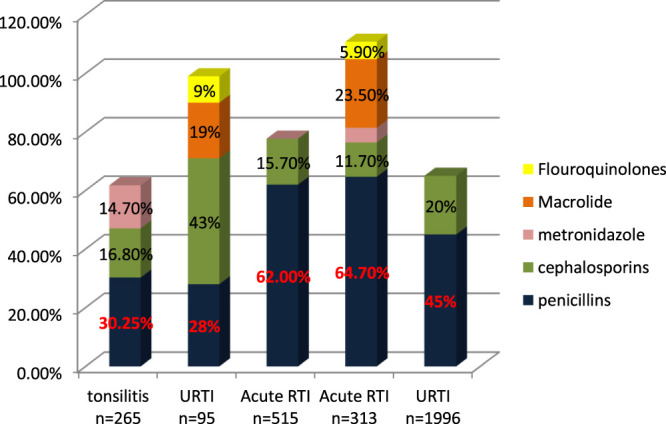
The statistical difference in antibiotic prescribing in different studies.

Antibiotics for the treatment of respiratory infection are commonly penicillins (amoxicillin and co-amoxiclav), cephalosporin (ceftriaxone), and macrolides. Penicillins are more often prescribed and recommended as shown in Figure 2. If penicillins are not effective and therapy fails, then ceftriaxone IV is recommended and has better results (Al Alawi et al., 2015).

#### Appropriate and Inappropriate Antibiotic Prescriptions in OPAT

To measure the appropriateness of prescriptions, different authors used different techniques. Al Salman et al. (2016) used a method to identify appropriateness in which prescriptions were studied and compliance checked with the ID department at MOH Bahrain or IDSA guidelines. They defined appropriateness as proper choice, frequency, duration, route, and combination, and inappropriate is defined as antibiotic without diagnosis or if drug choice, frequency, dose, route, or combination is improper in the emergency department. They found 81.9% (*n* = 1,313) (Al Salman et al., 2016) were inappropriate prescriptions, mostly males and in emergency departments. Alshakka et al. (2016) defined appropriateness with seven indicators; three core, one patient, and three complimentary, from the WHO drug use indicator (outpatient facilities) list were assessed, and they studied prescribing patterns in four hospitals in Yemen. They said, “Various indicators of antibiotics prescribing reveals that inappropriate prescribing of antibiotics is prevalent in Yemen regardless of public or private hospitals. No pharmacy and therapeutic committee to oversee the use and prescribing practices of antibiotics, and lack of practice of sensitivity testing before prescribing antibiotics contribute to inappropriate prescribing of antibiotics.” Butt et al. (2017) divided diagnosis into two types—1 in which antibiotics are indicated (bacterial infections) and diagnosis for which antibiotics are not indicated (viral infections)—through expert opinions, and they studied upper respiratory tract infection prescribing patterns of antibiotics in Qatar and found that 45% (*n* = 75,733) of antibiotics prescribed are deemed inappropriate least in the emergency department with 2% and were the highest for family physicians. Shaheen et al. (2018) measured appropriateness by comparing prescriptions with WHO guidelines on recommendation for acute respiratory tract infections in Saudi Arabia and stated, “Many physicians in Makkah Al Mukarramah are not following the WHO guidelines for Acute Respiratory Infection.” Ahmed et al. studied the outpatient prescribing pattern of cefuroxime, doxycycline, ciprofloxacin, and metronidazole in Saudi Arabia and concluded that a high percentage of antibiotics prescribed is inappropriate, and doxycycline is appropriately prescribed in the outpatients (Ahmed, 2020a; Ahmed, 2020b; Ahmed, 2020c; Ahmed, 2020d).

It is seen that antibiotics prescribed to outpatients in the Gulf region are inappropriate and need guidelines and further education for the physicians.

#### Penicillin Prescription in Outpatient and Emergency

Senok et al. (2009) studied the antibiotic-prescribing pattern for upper respiratory tract infections in Bahrain and found that penicillins (amoxicillin and co-amoxicalv) were 68/95 (71.58%) of all prescriptions. John et al. (2014) studied the antibiotic-prescribing pattern in acute tonsillitis in UAE and found that penicillins (co-amoxicalve) were 72/265 (27.1%) of all prescriptions. Al-Balushi et al. (2014) studied the antibiotic-prescribing trend in an Oman pediatric population and found that penicillins (amoxicillin, co-amoxiclav, and phenoxymethylpenicillin) were prescribed 34/48 (70.8%) times in outpatients. Al Salman studied the pattern of antibiotic prescribing in the emergency room in Bahrain and found that penicillins (amoxicillin, coamoxiclav, and cloxacillin) were prescribed 262/1,295 (20.23%) times in all prescriptions (Al Salman et al., 2016). Oqal et al. (2015) studied the antibiotic-prescribing pattern in an emergency department in Saudi Arabia and found that penicillins (amoxicillin and co-amoxiclav) were prescribed at 35.5%. Alshakka et al. (2016) studied the antibiotic-prescribing pattern in Yemen and found that penicillins (amoxicillin and penicillin) were prescribed 99/338 (29.3%) times in all prescriptions. Butt et al. (2017) studied the antibiotic-prescribing pattern in upper respiratory tract infections in Qatar and found that penicillins were 28% of all 75,733 claims. Shaheen et al. (2018) studied the antibiotic-prescribing pattern in acute respiratory tract infections in Saudi Arabia and found that penicillins (amoxicillin and co-amoxiclav) were prescribed 320/515 (62%) times in all prescriptions. Alanazi et al. (2018) studied community-acquired urinary tract infections in Saudi Arabia and found that penicillins (amoxicillin and co-amoxiclav) were prescribed for 26% of the total 1,449 patients with UTI. Alanazi et al. (2019) studied the antibiotic-prescribing pattern in an outpatient department in Saudi Arabia and found that penicillins (co-amoxiclav) were 32% of the total 3,872 antibiotic prescriptions.

**FIGURE 3:**
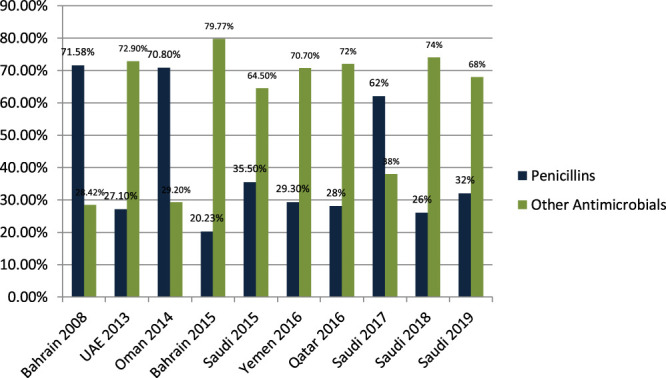
The difference of antibiotic prescribing in outpatients of the Gulf region among penicillins and other antimicrobials.

Results in Figure 3 shows a clear difference in prescribing patterns from 20.23% to a maximum of 71.58%. One of the most prescribed medications in the Gulf region is penicillin, especially Amoxicillin and Co-amoxiclav with some exceptions.

#### Cephalosporins Prescription in Outpatient and Emergency

Senok et al. (2009) studied the antibiotic-prescribing pattern for upper respiratory tract infections in Bahrain and found that cephalosporins (cefaclor, cefuroxime, cefprozil) were prescribed as 19/95 (20%) of all prescriptions. John et al. (2014) studied the antibiotic-prescribing pattern in acute tonsillitis in UAE and found that cephalosporins (cefpodoxime, cefidinir, ceftriaxone, cefuroxime, and cefexime) were prescribed as 107/265 (40.3%) of all prescriptions. Al-Balushi et al. (2014) studied the antibiotic-prescribing trend in an Oman pediatric population and found that cephalosporins (cefuroxime) were prescribed in 3/48 (6.25%) outpatients. Al Salman et al. (2016) studied the pattern of antibiotic prescribing in an emergency room in Bahrain and found that cephalosporins (cefuroxime and cephalexin) were prescribed as 616/1,295 (47.7%) of all prescriptions. Oqal et al. (2015) studied the antibiotic-prescribing pattern in an emergency department in Saudi Arabia and found that cephalosporins (cefprozole, cefuroxime, and cephalexin) were prescribed as 30.3%. Alsakka et al. (2016) studied the antibiotic-prescribing pattern in Yemen and found that cephalosporins (ceftriaxone, cefotaxime, cefixime) were prescribed as 132/338 (39%) of all prescriptions. Butt et al. (2017) studied the antibiotic-prescribing pattern in upper respiratory tract infections in Qatar and found that cephalosporins were 43% of all 75,733 claims. Shaheen et al. (2018) studied the antibiotic-prescribing pattern in acute respiratory tract infections in Saudi Arabia and found that cephalosporins (ceftriaxone, first and second generation) were prescribed as 116/515 (22.5%) of all prescriptions. Alanazi et al. (2018) studied community-acquired urinary tract infections in Saudi Arabia and found that cephalosporines were prescribed for 39% of the total 1,449 patients with UTI. Almasoudi et al. (2019) studied antimicrobial drug consumption in an ambulatory setting in Saudi Arabia and found that cephalosporins comprised 186/25,116 (0.74%) prescriptions as compared to all other antibiotics prescribed in units. Alanazi et al. (2019) studied the antibiotic-prescribing pattern in an outpatient department in Saudi Arabia and found that cephalosporins (ceftriaxone and cefuroxime) were 13% of the total 3,872 prescriptions for antibiotics.

**FIGURE 4:**
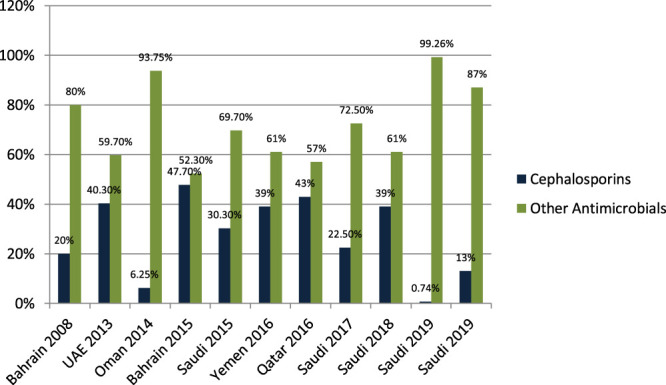
The difference in antibiotic prescribing in the Gulf region among cephalosporins and other antimicrobials.

Results in Figure 4 shows that cephalosporins are widely prescribed in outpatient settings in the Gulf region. Overall, 30%–40% of all antimicrobials prescribed in the Gulf region are cephalosporins with few exceptions.

#### Macrolide Prescribing in Outpatient and Emergency

Senok et al. (2009) studied the antibiotic-prescribing pattern for upper respiratory tract infections in Bahrain and found that macrolides (azithromycin and erythromycin) were prescribed as 8/95 (8.42%) of all prescriptions. John et al. (2014) studied the antibiotic-prescribing pattern in acute tonsillitis in UAE and found that macrolides (azithromycin, clarithromycin, and clindamycin) were prescribed as 73/265 (27.5%) of all prescriptions. Al-Balushi et al. (2014) studied the antibiotic-prescribing trend in an Oman pediatric population and found that macrolides (azithromycin) were prescribed for 11/48 (22.9%) of outpatients. Al Salman et al. (2016) studied the pattern of antibiotic prescribing in an emergency room in Bahrain and found that macrolides (clarithromycin and erythromycin) were prescribed 27/1,295 (2.08%) times in all prescriptions. Oqal et al. (2015) studied the antibiotic-prescribing pattern in an emergency department in Saudi Arabia and found that macrolides (azithromycin and clarithromycin) were prescribed as 20.9%. Alshakka et al. (2016) studied the antibiotic-prescribing pattern in Yemen and found that macrolides (azithromycin) were prescribed 21/338 (6.2%) times in all prescriptions. Butt et al. (2017) studied the antibiotic-prescribing pattern in upper respiratory tract infections in Qatar and found that macrolides were 19% of all 75,733 claims. Almasoudi (2019) studied antimicrobial drug consumption in an ambulatory setting in Saudi Arabia and found that macrolide prescriptions were 18,692/25,116 (74.4%) as compared to all other antibiotics prescribed in units. Alanazi et al. (2019) studied the antibiotic-prescribing pattern in an outpatient department in Saudi Arabia and found that macrolides (azithromycin) were 8% of the total 3,872 prescriptions of antibiotics.

**FIGURE 5:**
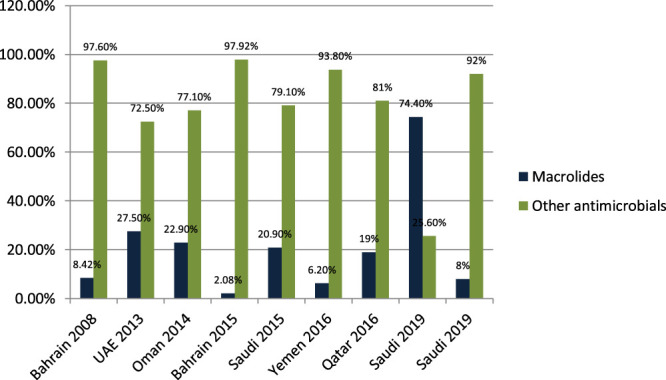
The difference of prescribing patterns of antimicrobials with macrolides.

It is evident from the results as shown in Figure 5 that macrolides are being prescribed highly in outpatient clinics and emergency departments in hospitals. On average, 18%–20% of all antimicrobials prescribed are macrolides. A study in Saudi Arabia in 2019 shows high consumption of azithromycin due to the per piece count. Variability is there, but on average, 17%–20% of prescribed drugs are macrolides.

#### Fluoroquinolones Prescribing in Outpatient and Emergency

John et al. (2014) studied the antibiotic-prescribing pattern in acute tonsillitis in UAE and found that fluoroquinolones (ciprofloxacin and levofloxacin) were prescribed 6/265 (2.2%) times of all prescriptions. Al-Salman et al. (2016) studied the pattern of antibiotic prescribing in an emergency room in Bahrain and found that penicillins fluoroquinolones (ciprofloxacin and norfloxacillin) were prescribed 274/1,295 (21.15%) times in all prescriptions. Oqal et al. (2015) studied the antibiotic-prescribing pattern in an emergency department in Saudi Arabia and found that fluoroquinolones (moxifloxacin and norfloxacillin) were 5% of prescriptions. Alsakka et al. (2016) studied the antibiotic-prescribing pattern in Yemen and found that fluoroquinolones (ciprofloxacin) were prescribed 23/338 (6.8%) times in all prescriptions. Butt et al. (2017) studied the antibiotic-prescribing pattern in upper respiratory tract infections in Qatar and found that fluoroquinolones were 9% of all 75,733 claims. Oqal (2015) studied community-acquired urinary tract infections in Saudi Arabia and found that fluoroquinolones were prescribed for 22% of the total 1,449 patients with UTI (Alanazi, 2018). Almasoudi et al. (2019) studied antimicrobial drug consumption in an ambulatory setting in Saudi Arabia and found that fluoroquinolones were 4774/25,116 (19%) prescriptions as compared to all other antibiotics prescribed in units. Alanazi et al. (2019) studied the antibiotic-prescribing pattern in an outpatient department in Saudi Arabia and found that fluoroquinolones (levofloxacin and ciprofloxacin) prescriptions were 19% of the total 3,872 antibiotic prescriptions.

**FIGURE 6:**
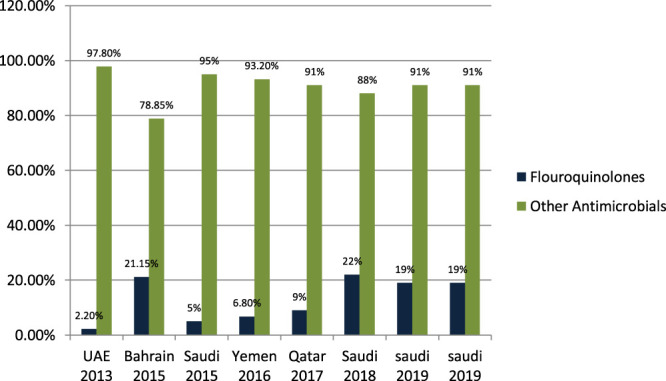
The difference in prescribing among fluoroquinolones and other antimicrobials in the Gulf region.

It is evident from the results as shown in Figure 6 that fluoroquinolones are being prescribed at around 12–15% in the Gulf region as compared to the other antimicrobials in outpatient antibiotic-prescribing patterns. This quantity may vary according to the type of infection but are overall 12–15%.

#### Nitromidazole Prescribing in Outpatient and Emergency

John LJ et al. (2014) studied the antibiotic-prescribing pattern in acute tonsillitis in UAE and found that nitromidazole (metronidazole and tinidazole) accounted for 38/265 (14.3%) of all prescriptions and metronidazole was prescribed 34 times in combination therapy. Alsalman et al. (2016) studied the pattern of antibiotic-prescribing in an emergency room in Bahrain and found that nitromidazole (metronidazole) was prescribed 101/1,295 (8.79%) times in all prescriptions. Alshakka et al. (2016) studied the antibiotic-prescribing pattern in Yemen and found that nitromidazole (metronidazole) was prescribed 41/338 (12.13%) times in all prescriptions. Alanazi et al. (2019) studied the antibiotic-prescribing pattern in an outpatient department in Saudi Arabia and found that nitromidazole (metronidazole) was 8% of the total 3,872 prescriptions of antibiotics. As is suggested, cephalosporins alone or in combination with metronidazole when anaerobes are implicated have the highest bacteriological and clinical efficacy (Pichichero, 2006).

**FIGURE 7:**
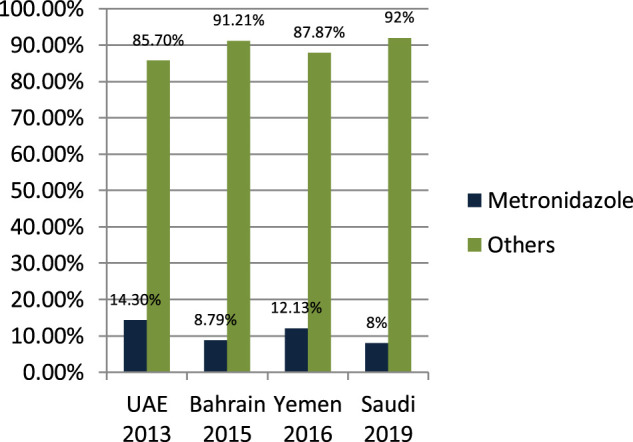
The difference among the prescribing of metronidazole and other antimicrobials.

It is clear from Figure 7 that metronidazole is being prescribed almost 10% of the time in outpatients of Gulf countries with other antimicrobials.

## Results and Discussion

Antibiotics for the treatment of respiratory infections are commonly penicillin (amoxicillin and co-amoxiclave), cephalosporin (ceftriaxone), and macrolides. The most common infection encountered in the outpatient departments is respiratory tract infection, out of which upper respiratory is more common than lower respiratory. Other infections encountered are urinary tract and gastric infections. Drugs prescribed for respiratory tract infections are from penicillin and cephalosporin to macrolide. Penicillins are more often prescribed and recommended as shown in Figure 2. If penicillins are not effective and therapy fails, then ceftriaxone IV is recommended and has better results (Senok et al., 2009; John et al., 2014; Al Alawi et al., 2015; Oqal et al., 2015; Shaheen et al., 2018; El-Gamal et al., 2020). If we closely observe Figure 2, it is clearly evident that penicillins are prescribed for respiratory tract infections to 60%. In some studies, it is observed that cephalosporins are prescribed up to a maximum of 50%, but overall, cephalosporins are prescribed second to penicillin, and macrolides are prescribed as a third option as they are not readily prescribed like the others. A study in the United States reveals that 37.3% of all patients in outpatient departments with respiratory infections were prescribed with antibiotics, and it varies with different sites (from 17.4 to 71%), and macrolides were the third most commonly prescribed in the Gulf region (Guzik et al., 2018). A study conducted in Spain reveals the percentage use of antibiotics in upper respiratory tract infections are, mostly amoxicillin prescribed in nonspecific URTIs (36%) and acute tonsillitis (47%), whereas cephalosporin is prescribed in acute otitis and sinusitis (20%) and macrolide 32% in nonspecific URTIs. In a study of antibiotic prescribing in outpatients for acute respiratory tract infections in influenza season a high rate of antibiotic prescribing in outpatients is observed even with laboratory-confirmed influenza (Havers et al., 2018).

It is seen that antibiotics prescribed in outpatients in the Gulf region are inappropriate and need guidelines and further education for the physicians (Al Salman et al., 2016; Alshakka et al., 2016; Butt et al., 2017; Shaheen et al., 2018). Results show a clear difference in the prescribing pattern of penicillin from a range of 20.23–71.58%. The data shows a clear difference of antibiotic-prescribing appropriateness from 20 to 70% roughly. We can expect a high rate of resistance and cost damage in the future due to these inappropriate prescriptions. Antibiotic prescribing assessed by the Global PPS system in 2017 found a very high number of prescribing errors that could be corrected with proper education and training of the healthcare staff involved in stewardship programs (Versporten et al., 2018). Prescribing practices can be assessed with WHO guidelines as in Namibia, where they were cross-checked with WHO indicators, and results were suboptimal (Niaz et al., 2019). Among the other suggestions to improve the appropriateness of prescribing, one can change through government policy as in the United States to improve the stewardship with the release of the National Strategy for Combating Antibiotic-Resistant Bacteria, the National Action Plan for Combating Antibiotic-Resistant Bacteria, and a Presidential Executive Order and more to make it government policy to find inappropriate prescribing and apply the policies through a national plan (Roberts et al., 2016). With a national plan, law enforcement can help to reduce the inappropriateness and resistance among antimicrobials as in Saudi Arabia, where inappropriateness decreases with law enforcement (Alrasheedy et al., 2020).

One of the most prescribed medications in the Gulf region is penicillin, in particular, Amoxicillin and Co-amoxiclave with some exceptions (Senok et al., 2009; John et al., 2014; Al-Balushi et al., 2014; Al Alawi et al., 2015; Al Salman et al., 2016; Oqal et al., 2015; Alshakka et al., 2016; Butt et al., 2017; Shaheen et al., 2018; Alanazi, 2018; Almasoudi et al., 2019; Alanazi et al., 2019). Penicillin and macrolide are the most common classes of antibiotics prescribed, and amoxicillin in combination with macrolide is prescribed as a single drug with higher rates in the United States (Cai and Zhou, 2017). Cephalosporins are widely prescribed in outpatient settings in the Gulf region. Overall, 30–40% of all antimicrobials prescribed in the Gulf region are cephalosporins with few exceptions (Senok et al., 2009; John et al., 2014; Al-Balushi et al., 2014; Al Alawi et al., 2015; al Salman et al., 2016; Oqal et al., 2015; Alshakka et al., 2016; Butt et al., 2017; Shaheen et al., 2018; Alanazi, 2018; Almasoudi et al., 2019; Alanazi et al., 2019; Ahmed, 2020c). Highly prescribed cephalosporins are ceftriaxone and cefuroxime. A study in China reveals that the most prescribed class of antibiotics is cephalosporin (Cai and Zhou, 2017) as compared to the Gulf region, where it is in second place. It is evident from the results of the research articles that macrolide is being prescribed highly in outpatient clinics and emergency departments of hospitals. On average, 18–20% of all antimicrobials prescribed is macrolide. A study in Saudi Arabia in 2019 shows high consumption of azithromycin due to the per piece count. Variation is there, but on average, 17–20% of prescribed drugs are macrolides (Senok et al., 2009; John et al., 2014; Al-Balushi et al., 2014; Al Alawi et al., 2015; Al Salman et al., 2016; Oqal et al., 2015; Alshakka et al., 2016; Butt et al., 2017; Shaheen et al., 2018; Alanazi, 2018; Almasoudi et al., 2019; Alanazi et al., 2019). Azithromycin is the most common macrolide prescribed in the Gulf region; others are clarithromycin and erythromycin, and one study shows the prescribing of clindamycin in outpatients. It is evident from the results that fluoroquinolones are being prescribed at around 12–15% in the Gulf region as compared to other antimicrobials in outpatient antibiotic-prescribing patterns. This quantity may vary according to the type of infection but overall are 12–15% (John et al., 2014; Al-Balushi et al., 2014; Al Alawi et al., 2015; Al Salman et al., 2016; Oqal et al., 2015; Alshakka et al., 2016; Butt et al., 2017; Shaheen et al., 2018; Alanazi, 2018; Almasoudi et al., 2019; Alanazi et al., 2019). A retrospective study of antibiotic prescription in Massachusetts reveals that the most prescribed antibiotic class is penicillin 215.6/1,000, then macrolide 177.1/1,000, quinolones 71.1/1,000, and cephalosporin 64.6/1,000 (Klevens et al., 2019). Ciprofloxacin is highly prescribed in the outpatient setting, especially for urinary tract infections, and others are levofloxacin and norfloxacin; the least prescribed is moxifloxacin. Another study in Mexico shows that quinolone is highly prescribed (27.7%) in outpatients; penicillins are 23.3%, cephalosporins are 17.3%, macrolides are 10.3%, but the overuse and subuse of antibiotics is very alarming (Sánchez-Huesca et al., 2020). It is clear from Figure 7 that metronidazole is being prescribed at almost 10% in outpatients of Gulf countries with other antimicrobials (John et al., 2014; Alshakka et al., 2016; Alanazi et al., 2019). Tinidazole is also prescribed but is the least common and rarely prescribed.

## Conclusion

It is suggested that antibiotic-prescribing patterns in outpatient and emergency departments in the Gulf region are inappropriate and need improvement, guidelines, and the education of physicians and patients, annual vaccination, and stewardship programs. Amoxicillin, co-amoxiclave, and cephalosporins are readily used for the treatment of respiratory tract infections. Penicillins are highly prescribed up to 50%, cephalosporins are up to 25–30% in overall prescriptions in outpatients in Gulf countries.

### Practice Implications

There is a need to reduce the higher rate of inappropriate prescribing of antibiotics in the Gulf region to reduce the upcoming resistance that will not only effect human life but the economy as well. There are some suggestions as to how to improve inappropriate prescribing, such as by routine vaccination for influenza, pneumococcal (Godman et al., 2020) and others. Use proper guidelines and continuously monitor the prescribing pattern with respect to the guidelines. Inappropriate prescribing can be discouraged by implementing antimicrobial stewardship in facilities, educating and awareness programs for the public to reduce self-medication, and testing to differentiate between viral and bacterial infections. An OECD report calculated that by investing as little as 2 U.S. dollars per person in a year could avoid 75% of death of people due to resistant microorganisms (Hofer, 2018). This will not only help to save lives but avoid the economic loss as well.

## Data Availability Statement

The raw data supporting the conclusion of this article will be made available by the authors, without undue reservation.

## Author Contributions

All authors listed have made a substantial, direct, and intellectual contribution to the work and approved it for publication.

## Conflict of Interest

The authors declare that the research was conducted in the absence of any commercial or financial relationships that could be construed as a potential conflict of interest.
